# Supplementary material for: The role of fMRI in the mind decoding process in adults: a systematic review
Source: PeerJ. 2025 Jan 17;13:e18795. doi: 10.7717/peerj.18795 (PMC11745131; doi:10.7717/peerj.18795)
Supplement: Supplemental Information 1 [file peerj-13-18795-s001.doc]

Dear Editor,

Thank you for the insightful comments and the opportunity to improve our manuscript. In response to the request for clarification regarding the intended audience for our systematic review, we have provided the following information:

Our systematic review is primarily aimed at the following audiences:

- Neuroscientists and Cognitive Psychologists: Researchers interested in the neural mechanisms underlying cognitive processes, particularly those involved in mind reading and social cognition.
- Clinicians and Mental Health Professionals: Professionals who may apply insights from this research in clinical settings, especially in diagnosing and treating neurological and psychiatric conditions.
- Educators and Students: Those teaching or studying neuroscience, psychology, or related fields who can benefit from a comprehensive overview of current findings and methodologies in this area.

We believe that this review will provide valuable insights and foster further research and application within these fields.

Thank you for considering our revisions, and please let us know if there are any further changes required.

Best regards,

Dr. Khandaker
